# Four Novel Zn (II) Coordination Polymers Based on 4′-Ferrocenyl-3,2′:6′,3′′-Terpyridine: Engineering a Switch from 1D Helical Polymer Chain to 2D Network by Coordination Anion Modulation

**DOI:** 10.3390/ma10121360

**Published:** 2017-11-27

**Authors:** Lufei Xiao, Dajun Wu, Xuchun Wang, Wei Du, Jun Zhang, Shengli Li, Hongping Zhou, Jieying Wu, Yupeng Tian

**Affiliations:** 1 Department of Chemistry, Key Laboratory of Chemistry for Inorganic/Organic Hybrid Functionalized Materials of Anhui Province, Anhui University, Hefei 230601, China; xlf3345@163.com (L.X.); djwu0926@163.com (D.W.); wangxc@ahstu.edu.cn (X.W.); duwei1247@163.com (W.D.); zhangjun@ahjzu.edu.cn (J.Z.); lsl1968@ahu.edu.cn (S.L.); zhpzhp@263.net (H.Z.); 2 Department of Food and Environmental Engineering, Chuzhou Vocational and Technical College, Chuzhou 239000, China; 3 State Key Laboratory of Coordination Chemistry, Nanjing University, Nanjing 210093, China

**Keywords:** coordination polymer, 3,2′:6′,3′′-terpyridine, Zn^II^ ion, crystal structure, coordination anion

## Abstract

Four novel Zn^II^ coordination polymers, [(ZnCl_2_)_2_(L)_2_]_n_ (**1**), [(ZnBr_2_)_2_(L)_2_]_n_ (**2**), and [(ZnI_2_)_2_(L)_2_]_n_ (**3**) and {[Zn(SCN)_2_]_1.5_(L)_3_}_n_ (**4**), have been synthesized based on 4′-ferrocenyl-3,2′:6′,3′′-terpyridine with Zn^II^ ions and different coordination anions under similar ambient conditions. Their structures have been confirmed using single crystal X-ray diffraction analysis, showing that complexes **1**–**3** are one-dimensional (1D) double-stranded metal ion helical polymer chains and complex **4** is of a two-dimensional (2D) network. The structural transformations of them from a 1D polymer chain to a 2D network under the influence of the coordination anions has been systematic investigated. Furthermore, the optical band gaps have been measured by optical diffuse reflectance spectroscopy, revealing that the ligand and the complexes should have semiconductor properties.

## 1. Introduction

**Figure 1:**
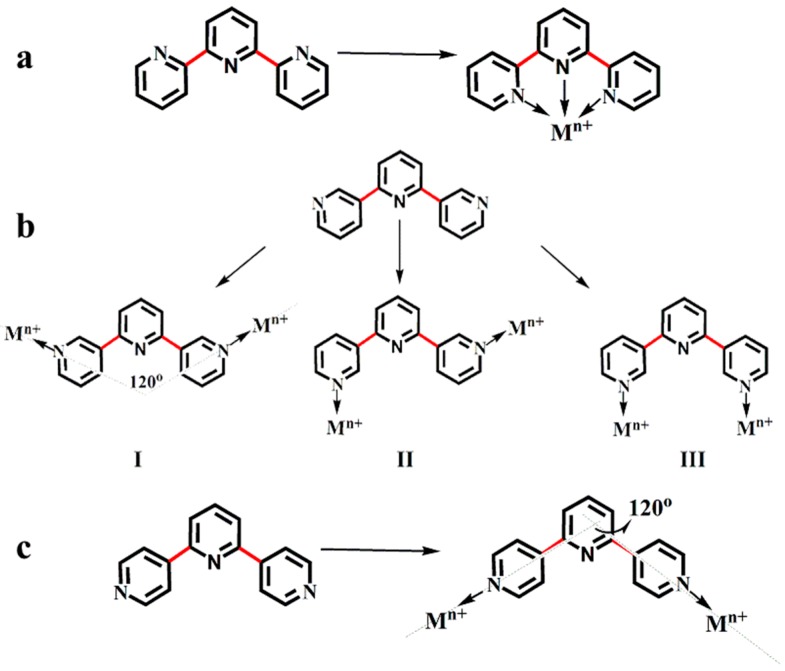
Three types of terpyridine: 2,2′:6′,2′′-terpyridine. (**a**) 3,2′:6′,3′′-terpyridine; (**b**) 4,2′:6′,4′′-terpyridine; (**c**) the directional metal-binding properties of them.

Terpyridines, which are oligopyridines, represent a very popular and versatile building block of ligands for the constructing functional complexes [1]. Terpyridines possess 48 isomers, of which the 2,2′:6′,2′′-terpyridine, 3,2′:6′,3′′-terpyridine and 4,2′:6′,4′′-terpyridine (Figure 1) are well known, above all because of their ease of synthesis and functionalization in the 4′-position [1,2,3,4,5,6].

In the three types of terpyridine, 2,2′:6′,2′′-terpyridine possesses bis-chelating ability due to inter-ring C–C bond rotation (in Figure 1a) and has been easily functionalized. Since 2,2′:6′,2′′-terpyridine was first isolated by Morgan and Burstall in the 1930s, it has become a famous building block of functional ligands [3,7,8]. Its complexes have been wide applications in material fields. Later, 3,2′:6′,3′′-terpyridine and 4,2′:6′,4′′-terpyridine were reported in 1995, by Butler [9] and in 1998, by Garmendia [10], respectively. The two type terpyridine ligands can coordinate with metal ions in the outer pyridine rings, while the central pyridine rings are usually not involved in coordination [2,11]. The inter-ring C–C bond rotations have no effect on the ligand conformer (Figure 1c), and therefore 4,2′:6′,4′′-terpyridine, which acts as an excellent V-shaped building block, has received increasing attention [2,4,5,12]. There are over 200 types of complexes, which have been reported with this type of terpyridine in coordination polymers and networks etc. [2,4,5,12].

Aside from the above two isomers, when this type of ligand coordinates with metal ions, 3,2′:6′,3′′-terpyridine can form three binding modes (*trans-trans*, *cis-trans*, and *cis-cis*) due to the inter-ring C–C bond rotations (Figure 1b). This leads to difficulties in predicting the structures of the complexes with this type of ligand [2,11]. To date, the complexes containing this type of ligand have rarely been explored, with only more than twenty complexes reported in total [2,13]. In particular, more efforts should be made to search for functionalized 3,2′:6′,3′′-terpyridine ligands in the field of coordination chemistry and materials [4,13,14,15,16,17,18,19].

**Figure 2:**
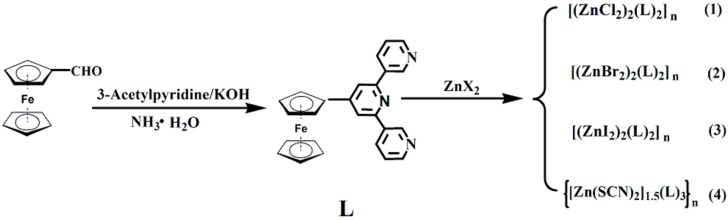
Synthetic routes for the ligand and complexes **1**–**4**.

Based on the aforementioned considerations, 4′-ferrocenyl-3,2′:6′,3′′-terpyridine and ZnX_2_ (X = Cl, Br, I, and SCN) have been selected as the ligand and the metal ion nodes, and four novel complexes, [(ZnCl_2_)_2_(L)_2_]_n_ (**1**), [(ZnBr_2_)_2_(L)_2_]_n_ (**2**) and [(ZnI_2_)_2_(L)_2_]_n_ (**3**), and {[Zn(SCN)_2_]_1.5_(L)_3_}_n_ (**4**), have been synthesized under similar conditions, as shown in Figure 2. Their structural features have been systematically investigated, and their structural transformations from one-dimensional (1D) polymer chain to two-dimensional (2D) network under the influence of the coordination anions have been studied. In addition, the optical band gaps of the ligand and the complexes have been confirmed by the Kubela-Munk-transformed diffuse reflectance spectra.

## 2. Experimental Section

### 2.1. Materials and Methods

Ferrocenecarboxaldehyde was synthesized according to a reported method [20]. All reagents and solvents were obtained commercially, and purified according to the standard method. FT-IR spectra were recorded with the solid (KBr pellets) using a NEXUS-870 (Thermo Nicolet Corporation, Denver, CO, USA) spectrophotometer in the range of 400–4000 cm^−1^. Elemental analyses (C, H, and N) were performed using a Perkin-Elmer 240 analyzer (PerkinElmer Corporation, Waltham, MA, USA). The ^1^H and ^13^C NMR spectra were recorded on a Bruker Avance 400 spectrometer (Bruker Corporation, Karlsruhe, Germany) at 25 °C, and the chemical shifts were reported as parts per million (ppm) from Tetramethylsilane (TMS). Coupling constants *J* were given in hertz. Mass spectra were recorded on a Bruker Autoflex III SMartbeam instrument (MALDI-TOF, Bruker Corporation, Larlsruhe, Germany). The solid-state Ultraviolet-Visible (UV-vis) diffuse reflectance spectra were recorded at room temperature on a U-4100 Spectrometer (Hitachi Corporation, Tokyo, Japan) in the wavelength range of 200–900 nm. The instrument was equipped with an integrating sphere and controlled by a personal computer. The samples were ground into fine powder and pressed onto a thin glass slider holder. BaSO_4_ plate was used as a standard (100% reflectance).

### 2.2. X-ray Crystallography

The single-crystal X-ray diffraction measurements of the ligand and complexes **1**–**4** were carried out on a Siemens Smart 1000 CCD diffractometer (Siemens Corporation, Munich, Germany) at room temperature, and the determination of unit cell parameters and data collections were performed with Mo-K*α* radiation (λ = 0.71073 Å). Unit cell dimensions were collected with least-squares refinements and all structures were solved by direct methods using SHELXS-97. The other non-hydrogen atoms were located in successive difference Fourier syntheses. The final refinement was performed using full-matrix least-squares methods with anisotropic thermal parameters for non-hydrogen atoms on *F*^2^. The hydrogen atoms were added theoretically and riding on the concerned atoms. Crystal data and structural refinement parameters for **L** and complexes **1**–**4** have been summarized in Table 1.

### 2.3. Synthesis and Characterization of Compounds

1. 4′-Ferrocenyl-3,2′:6′,3′′-Terpyridine (**L**)

3-Acetylpyridine (4.84 g, 40.0 mmol) was added to a solution of ferrocenecarboxaldehyde (4.28 g, 20.0 mmol) in EtOH (20 mL). KOH pellets (2.24 g, 40.0 mmol) were added to the reaction mixture in one portion, then the mixture was stirred at room temperature for several minutes, followed by NH_3_·H_2_O (25%, 48 mL). After 12 h of reflux, the precipitate was collected by filtration, washed with water and EtOH, and recrystallized from EtOH. The red solid was isolated and dried in vacuo, weighing 3.95 g (47.2%). ^1^H NMR (400 MHz, DMSO, ppm): δ 9.50 (s, 2H), 8.68 (d, *J* = 7.8 Hz, 4H), 8.17 (s, 2H), 7.59 (dd, *J* = 7.5 Hz, 5.0, 2H), 5.31 (s, 2H), 4.56 (s, 2H), 4.11 (s, 5H). ^13^C NMR (100 MHz, DMSO, ppm): 153.82, 151.39, 149.98, 148.03, 134.23, 133.98, 123.78, 116.24, 70.29, 69.81, 67.43. IR (cm^−1^): 3075 (m), 3036 (m), 1603 (vs), 1572 (s), 1545 (s), 1476 (v), 1447 (m), 1424 (s), 1403 (s), 1383 (s), 1105 (s), 1059 (m), 1009 (s), 823 (s), 762 (m), 487 (s). MALDI-TOF-MS: *m/z*, 416.198 [M^+^]. Anal. Calcd. for C_25_H_19_FeN_3_: C, 71.960; H, 4.590; N, 10.070. Found: C, 72.198; H, 4.559; N, 10.108.

2. [(ZnCl_2_)_2_(L)_2_]_n_ (**1**)

A solution of **L** (0.052 g, 0.125 mmol) in CHCl_3_ (5 mL) was placed in a test tube (25 mL). MeOH (10 mL) was layered on the top of this solution, followed by a solution of ZnCl_2_ (0.017 g, 0.125 mmol) in MeOH (5 mL). The test tube was sealed with a glass stop and left to stand at room temperature about 10 days. Red crystals, suitable for single crystal X-ray diffraction analysis, formed on the glass wall. The crystals were collected by decanting the solvent and washed with H_2_O and MeOH, respectively, and dried in vacuo, weighting 0.049 g (67.9%, based on **L**, similarly hereinafter). IR (cm^−1^): 3089 (m), 2924 (m), 1610 (vs), 1544 (s), 1481 (s), 1409 (s), 1388 (s), 1256 (m), 1196 (s), 1107 (s), 1033 (s), 1018 (m), 874 (m), 753 (s), 497 (s). Anal. Calcd. for C_50_H_38_Cl_4_Fe_2_N_6_Zn_2_: C, 54.240; H, 3.460; N, 7.590. Found: C, 53.900; H, 3.460; N, 7.555.

3. [(ZnBr_2_)_2_(L)_2_]_n_ (**2**)

The same procedure as that for **1** was used except that ZnCl_2_ was replaced by ZnBr_2_ (0.028 g, 0.125 mmol), red crystals were obtained, weighting 0.055 g (65.0%). IR (cm^−1^): 3091 (m), 2924 (m), 1609 (s), 1544 (s), 1481 (s), 1438 (m), 1408 (s), 1387 (m), 1323 (m), 1197 (m), 1107 (s), 1056 (s), 1018 (s), 872 (s), 770 (m), 504 (s). Anal. Calcd. for C_50_H_38_Br_4_Fe_2_N_6_Zn_2_: C, 46.740; H, 2.980; N, 6.540. Found: C, 46.980; H, 2.962; N, 6.582.

4. [(ZnI_2_)_2_(L)_2_]_n_ (**3**)

The same procedure as that for **1** was used except that ZnCl_2_ was replaced by ZnI_2_ (0.040 g, 0.125 mmol), red crystals were obtained, weighting 0.059 g (64.1%). IR (cm^−1^): 3089 (w), 2924 (m), 1608 (vs), 1544 (vs), 1482 (s), 1436 (s), 1408 (s), 1387 (s), 1323 (s), 1255 (s), 1196 (s), 1106 (s), 1033 (s), 1000 (m), 815 (s), 770 (s), 502 (s). Anal. Calcd. for C_50_H_38_Fe_2_I_4_N_6_Zn_2_: C, 40.770; H, 2.600; N, 5.710. Found: C, 41.050; H, 2.581; N, 5.761.

5. {[Zn(SCN)_2_]_1.5_(L)_3_}_n_ (**4**)

The same procedure as that for **1** was used except that ZnCl_2_ was replaced by Zn(SCN)_2_ (0.023 g, 0.125 mmol), red crystals were obtained, weighting 0.050 g (63.8%). IR (cm^−1^): 3079 (m), 2955 (m), 2077 (vs), 2062 (s), 1602 (vs), 1545 (s), 1478 (m), 1430 (m), 1409 (m), 1385 (m), 1321 (m), 1191 (s), 1135 (s), 1108 (s), 1032 (m), 1008 (m), 877 (s), 751 (s), 496 (s). Anal. Calcd. for C_156_H_114_Fe_6_N_24_S_6_Zn_3_: C, 61.470; H, 3.770; N, 11.030. Found: C, 60.960; H, 3.736; N, 10.980.

## 3. Results and Discussion

### 3.1. Syntheses

The syntheses are summarized in Figure 2. The ligand was synthesized using the one-pot method starting from ferrocenecarboxaldehyde and 3-Acetylpyridine in EtOH in the presence of KOH and NH_3_ in a similar way to the reported work [11,18,21]. The crude product was purified by recrystallization from EtOH, The ligand was prepared with satisfactory yield of 47.2% and systematically characterized with ^1^H/^13^C NMR, FI-IR spectrum, mass spectrum and elemental analysis. The crystal of the ligand was grown from a DCM/Benzene solution of the compound by slow evaporation. The preparations of complexes **1**–**4** were performed in a similar way to reported work [11,18,21] by layering of MeOH and CHCl_3_ solution of ZnX_2_ and the ligand, respectively, and their crystals were formed in about 10 days in high yields.

### 3.2. Description of Crystal Structures

#### 3.2.1. Structure of the Ligand

**Figure 3:**
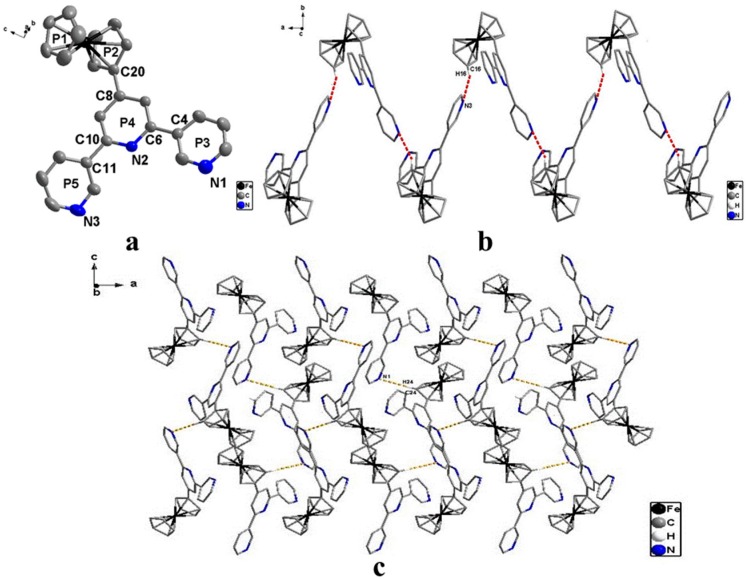
The structure of the ligand. (**a**) Atom part numbering and ring labelling (H atoms are omitted for clarity); (**b**) One-dimensional (1D) chain interacted through C–H···N (red dotted lines) of **L** (along the *a* axis); (**c**) Two-dimensional (2D) networks interacted through C–H···N (yellow dotted lines) of **L** (along the *c* axis).

The X-ray structural analysis shows that the ligand crystallizes in the orthorhombic chiral space group P2_1_2_1_2_1_, and the molecular structure of **L** is illustrated in Figure 3. Although the crystal structure of **L** has previously been reported [18], herein, we compare the ligand with the other compounds, 4′-ferrocenyl-2,2′:6′,2′′-terpyridine [22,23] and 4′-ferrocenyl-4,2′:6′,4′′-terpyri-dine [21], in detail, based on the structural re-analysis. As shown in Figure 3, the two cyclopentadienyl rings of the ferrocenyl moiety are nearly parallel, and the dihedral angle between P1/P2 is 1.532°, and which is smaller than that of the 4′-ferrocenyl-2,2′:6′,2′′-terpyridine (1.864°) [22,23] and 4′-ferrocenyl-4,2′:6′,4′′-terpyridine (1.889°) [21]. The 3,2′:6′,3′′-terpyridine group adopts the *cis*-*cis* conformation about the interannular C–C bond which is dissimilar to the *trans*-*trans* conformation of 4′-ferrocenyl-2,2′:6′,2′′-terpyridine [22,23]. It is not completely coplanar, and the dihedral angles between P3/P4 and between P4/P5 are 1.908° and 35.331°, respectively. This shows that the 3,2′:6′,3′′-terpyridine group coplanarity is weaker than that of the 2,2′:6′,2′′-terpyridine group (1.1° and 13.5°) in the 4′-ferrocenyl-2,2′:6′,2′′-terpyridine [22,23] and that of the 4,2′:6′,4′′-terpyridine group (5.368° and 5.705°) in the 4′-ferrocenyl-4,2′:6′,4′′-terpyridine [21]. The directly bonded cyclopentadienyl ring (P2) of the ferrocenyl group is twisted about the C8–C20 bond, resulting in a dihedral angle of 28.650° with the central pyridine ring (P4) of the 3,2′:6′,3′′-terpyridine group, and the dihedral angle is greater than that of the 4′-ferrocenyl-2,2′:6′,2′′-terpyridine (19.000°) [22,23] and the 4′-ferrocenyl-4,2′:6′,4′′-terpyridine (21.651°) [21]. The bond lengths of C20–C8, C4–C6 and C10–C11 are 1.463, 1.486 and 1.474 Å, respectively, which are similar to those of the 4′-ferrocenyl-2,2′:6′,2′′-terpyridine (1.472, 1.486, and 1.484 Å) [22,23] and the 4′-ferrocenyl-4,2′:6′,4′′-terpyridine group (1.486, 1.495, and 1.486 Å) [21] and are obviously shorter than the normal C–C bond length. There is the conjugation effect in the ligand molecule, similar to the 4′-ferrocenyl-2,2′:6′,2′′-terpyridine and the 4′-ferrocenyl-4,2′:6′,4′′-terpyridine group. 

A cyclopentadienyl ring interacts with a pyridine ring in a neighboring molecule through C–H···N interaction to yield a 1D “*zigzag*” chain structure, in which the formation of the 1D chain is similar with that of the 4′-ferrocenyl-4,2′:6′,4′′-terpyridine [21]. The C16–H16 and H16···N3 bond lengths are 0.907 and 2.667 Å, and the angle of C16–H16···N3 is 156.77°. The 1D chain forms 2D structures through C–H···N interactions, and the C24–H24 and H24···N1 bond lengths are 0.941 and 2.729 Å and the angle of C16–H16···N3 is 170.08°.

#### 3.2.2. Structure of Complexes **1**–**4**

1. One-Dimensional Polymer Chains

[(ZnCl_2_)_2_(L)_2_]_n_ (**1**), [(ZnBr_2_)_2_(L)_2_]_n_ (**2**), and [(ZnI_2_)_2_(L)_2_]_n_ (**3**).

The X-ray structural analysis reveals that complexes **1**–**3** all feature 1D polymer chains and complexes **1**–**3** all crystallize in the same triclinic space group *Pī* with similar cell parameters. Housecroft and co-workers [18] recently reported the reactions of the ligand with ZnCl_2_ and ZnBr_2_ to obtain the melallosqure [{ZnCl_2_(L)}_4_·3CHCl_3_·3MeOH] and 1D helical polymer [{ZnBr_2_(L)}·MeOH]_n_. However, crystal growth of the two reactions under analogous conditions resulted in the 1D helical polymers [(ZnCl_2_)_2_(L)_2_]_n_ and [(ZnBr_2_)_2_(L)_2_]_n_ in our works, and the structure of [(ZnBr_2_)_2_(L)_2_]_n_ is different from that of [{ZnBr_2_(L)}·MeOH]_n_. Furthermore, the reaction of the ligand with ZnI_2_ has also led to 1D helical polymer [(ZnI_2_)_2_(L)_2_]_n_.

**Figure 4:**
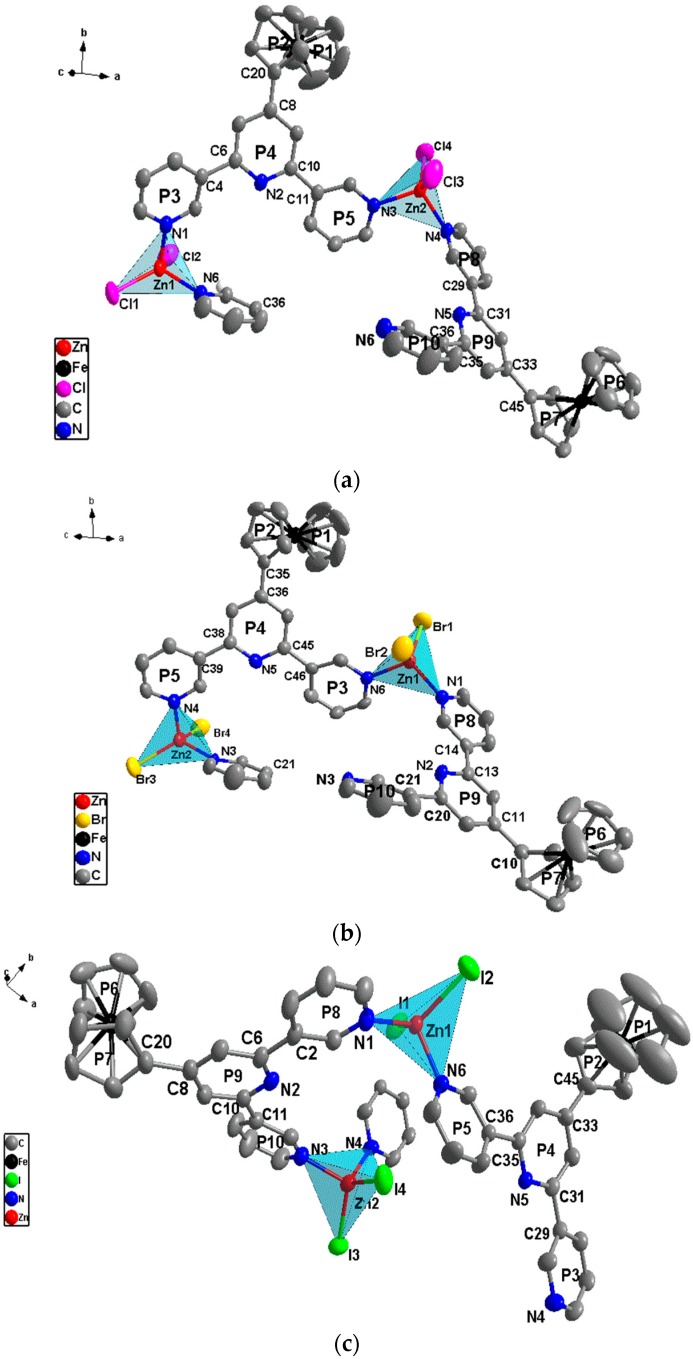
The coordination environment of the Zn^II^ ion in complexes **1** (**a**), **2** (**b**) and **3** (**c**) with atom part numbering and ring labeling (H atoms and solvent molecules are omitted for clarity).

**Figure 5:**
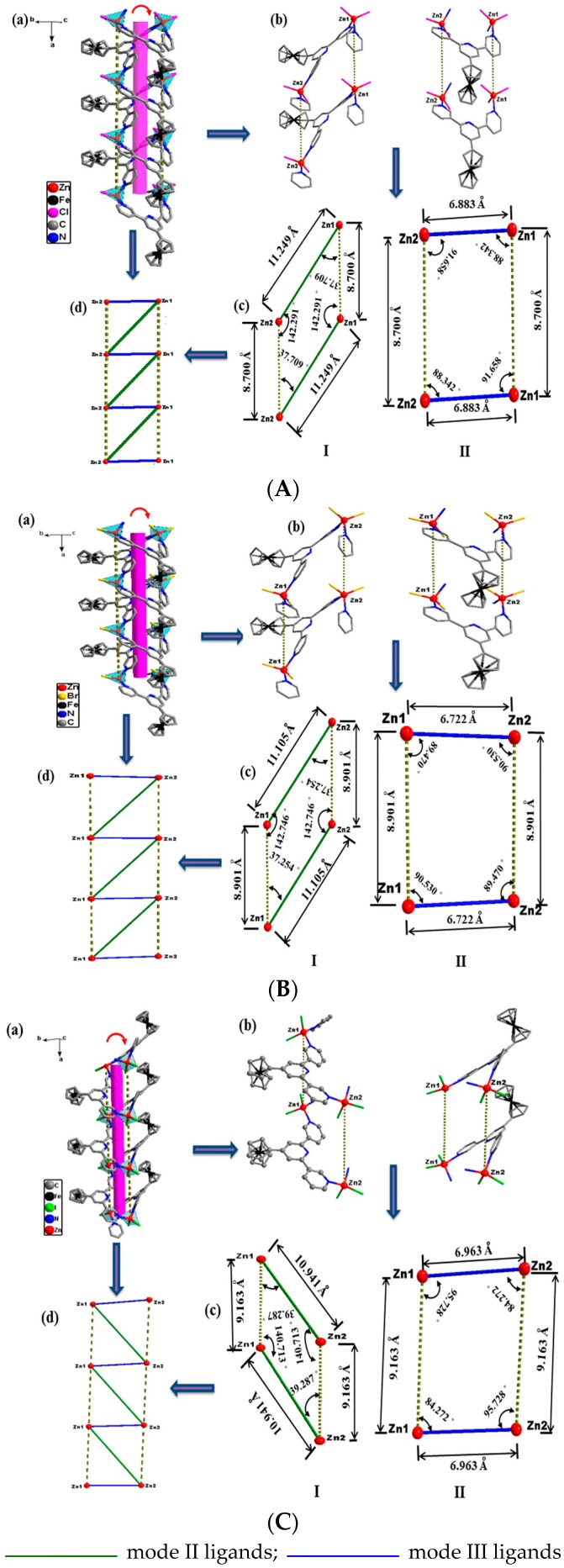
(a) The 1D double-stranded metal ion left-handed helical polymer chains of complexes **1** (**A**), **2** (**B**) and **3** (**C**) (H atoms and solvent molecules are omitted for clarity); (b) two connecting formations connected by different coordination mode ligands; (c) two types of parallelogram-like skeletons; (d) the ladder-like skeleton.

As shown in Figure 4, the asymmetric unit in the three complexes consists of two independent Zn^II^ ions and two ligands, and the Zn^II^ ion is tetra-coordinated with a slightly distorted tetrahedral geometry. Two coordination sites are occupied by two outer-pyridyl N atoms from different ligands and the other two coordination sites are occupied by halogen atoms (Cl, Br or I). The selected bond parameters of the three complexes are listed in Table S1. The central pyridiyl N atom of the ligand is not coordinated with the metal ion, just as the described above, and non-coordination of the central pyridine ring is typical of the 3,2′:6′,3′′-terpyridine and its derivatives [2,11,18]. Of the three possible conformations shown in Figure 1b, it is interesting that the ligands in the three complexes adopt two different binding modes, *cis*-*trans* (mode II) and *cis*-*cis* (mode III), for which there is only one binding mode (*cis*-*trans*) in the complex of the ZnBr_2_ with the ligand in Housecroft’s works [18]. Compared with the free ligand, the distortion of the two modes of ligands in the three complexes all have changes of various degrees. In particular, the mode II ligands distorted largely, and the collection distances of rings and dihedral angles of complexes **1**–**3** are listed in Table S2. The ligands act as two types of bridges that connect the Zn^II^ ions to form 1D double-stranded metal ion left-handed helical polymer chains along the *a* direction, as shown in Figure 5a.

Due to the two binding modes of ligands, it is observed that there are two types of connection formations (**I** and **II**) in the three complexes, which is shown in Figure 5b. As shown in Figure 5c, the two connections form two types of parallelogram-like frameworks and the four Zn^II^ ions sit at the vertices of four-sided parallelogram, which makes complexes **1**–**3** form the ladder-like skeleton shown in Figure 5d. Notice from Figure 5c that halogen anion ligands are in *trans* form horizontally but are *cis* form vertically. The horizontal and longitudinal distances of Zn···Zn are mostly affected by the structural changes of the ligands, and the halogen anion ligands have a significant influence on the longitudinal distances of Zn···Zn. As shown in Figure 5 c, the distances of Zn1···Zn2 are 11.249 (**1**), 11.105 (**2**) and 10.941 (**3**) Å in formation **I** and 6.883 (**1**), 6.722 (**2**) and 6.963 (**3**) Å in formation **II**. The longitudinal Zn1···Zn1 and Zn2···Zn2 distances are 8.700 (**1**), 8.901 (**2**) and 9.163 (**3**) Å, respectively. The horizontal distances of Zn···Zn of the two formations in the three complexes gradually become shorter, but the longitudinal distances of which become longer with the halogen atoms changing (Cl, Br, and I), which are the anion ligands for these complexes. Furthermore, the distance of Zn1···Zn2 in formation **II** of complex **3** is a little longer than that of complexes **1** and **2**. As shown in Figure 5b and Table S2, the two connecting formations of complexes **1** and **2** are very similar and the distortion of the two binding modes of ligands in complex **2** is a little greater than that of complex **1**. However, the two connecting formations of complex **3** are different from those of complexes **1** and **2** and the distortion of the two binding modes of ligands is also distinct to that of complexes **1** and **2**, which makes the ferrocene group orientations of the two binding modes of ligands in complex **3** in opposite direction to those of complexes **1** and **2**, shown in Figure 5a.

2. Two-Dimensional Network Polymer

{[Zn(SCN)_2_]_1.5_(L)_3_}_n_ (**4**)

**Figure 6:**
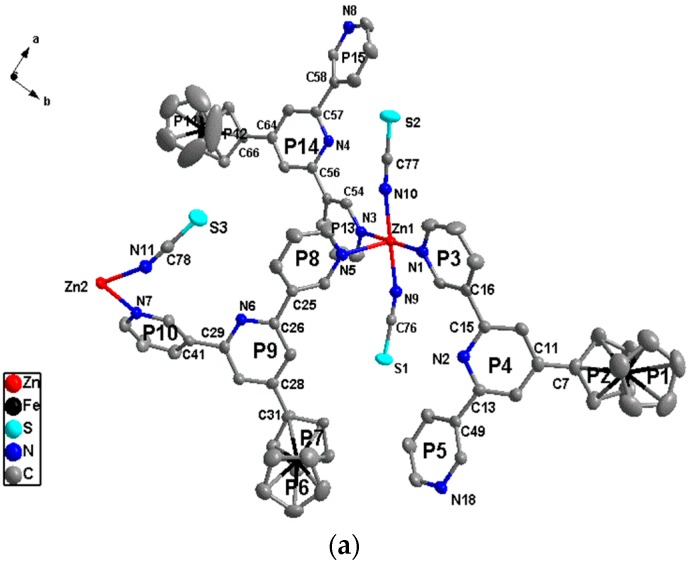

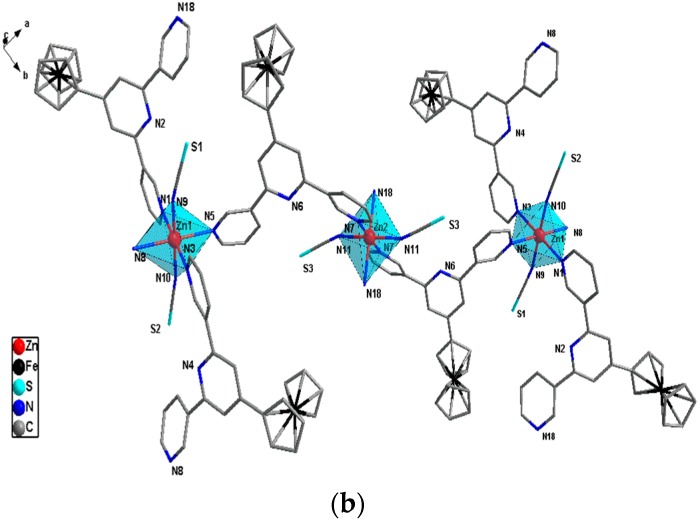
The asymmetric unit (**a**) and coordination environment of the Zn^II^ ion (**b**) in complex **4** with atom part numbering and ring labeling (H atoms and solvent molecules are omitted for clarity).

**Figure 7:**
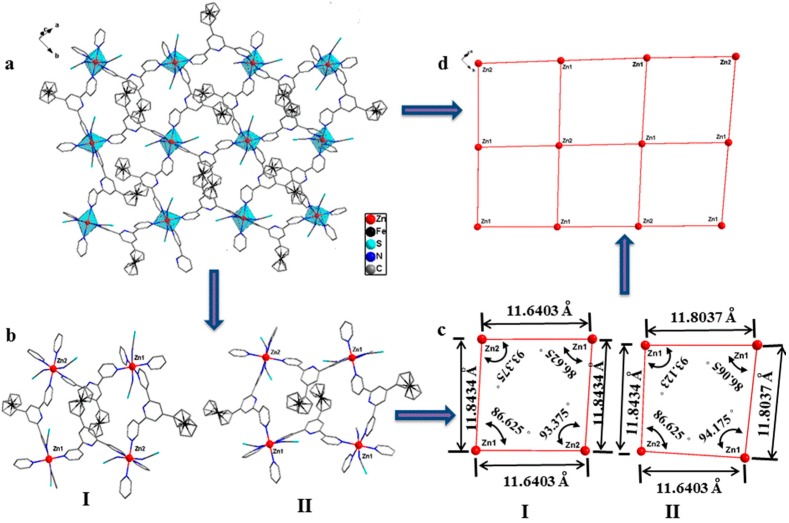
(**a**) The 2D network polymer of complex **4** (H atoms and solvent molecules are omitted for clarity); (**b**) two modes of hole-structure unit; (**c**) two types of quadrangle; (**d**) the 2D network.

The result of the structure determination reveals that the complex crystallizes in the monoclinic P2_1_/n and confirms that {[Zn(SCN)_2_]_1.5_(L)_3_}_n_ (**4**) is of a 2D network coordination polymer. As shown in Figure 6, the asymmetric unit of complex **4** contains one and a half Zn(SCN)_2_ and three ligands, and the Zn^II^ ion is hexa-coordinated with a slightly distorted octahedral geometry. The selected bond parameters of this complex are listed in Table S3. In the complex, each hexa-coordinated Zn^II^ ion connects with four outer-pyridyl N atoms from four disparate ligands and two N atoms from two disparate anionic groups (SCN). It is similar to complexes **1**–**3** that the central pyridiyl N atoms of ligands have not participated in coordination with metal ion, but the ligands only adopt one *cis*-*trans* (mode II) binding mode coordinated with the Zn^II^ ion. They have three different structural parameters in this complex. The complex with a 2D network polymer, shown in Figure 7, has been constructed by the three structural parameters ligands. Compared with the free ligand and complexes **1**–**3**, the ligand molecules have more distortions, and the collection distances of rings and dihedral angles are listed in Table S2. 

As shown in Figure 7, Zn^II^ ions are almost on a straight line along horizontal and vertical directions in the complex, and are arranged in the order of ···Zn2···Zn1···Zn1···Zn2···. There are two modes of hole-structure units, I and II, in the polymeric network. Furthermore, two modes of hole-structures form two types of quadrangle, I and II, and quadrangle I is approximately a parallelogram. Two types of quadrangle construct the network and arrange in the order of ···I···II···II···I··· along horizontal and vertical directions in the network.

3. Structural Comparison and Analysis

As shown Tables S1 and S3, Zn-N (N is from outer-pyridine of ligands) bond distances lie between 2.046 and 2.063 (**1**), 2.053 and 2.088 (**2**), 2.042 and 2.070 (**3**), 2.249 and 2.309 (**4**) Å, respectively. Zn-X (X = Cl, Br and I) and Zn-N (N is from the isothiocyanate radical) bond distances are in the range of 2.1983 to 2.2178 (**1**), 2.3443 to 2.3591 (**2**), 2.5322 to 2.5547 (**3**), 2.058 to 2.079 (**4**) Å, respectively. It is worth noting that anion ligands are mono-atom in complexes **1**–**3** but multi-atoms in complex **4**. Although the bond length of Zn-N (N is from the isothiocyanate radical) is shorter than that of Zn-X, the distance between the Zn ion with the centroid of anion ligands in complex **4** is obviously longer than that of complexes **1**–**3**. Because the larger anion ligands have been introduced, which results in larger space resistances in complexes **1**–**4**, there is a regular change with a gradual increase in the Zn-N bond distances and the lengths between the Zn ions with the anion ligands in the four complexes.

As shown in Figure 5 and Figure 7, the horizontal Zn···Zn distances of the two types of parallelogram-like in complexes **1**–**3** and two types of quadrangle in complex **4** are 11.249 and 6.883 (**1**), 11.105 and 6.722 (**2**), 10.941 and 6.963 (**3**), and 11.6403 and 11.8037 (**4**) Å, and the longitudinal Zn···Zn distances are 8.700 (**1**), 8.901 (**2**), 9.163 (**3**), 11.8434 and 11.8037 (**4**) Å, respectively. In the longitudinal variation of the complexes **1**–**3**, anion ligands are *cis* form which results in steric hindrance, so the longitudinal Zn···Zn distances gradually increase with the anion ligands from Cl to I. However, in the horizontal variation of these three complexes, anion ligands are of the *trans* form, and the horizontal Zn···Zn distances are mostly affected by the ligand configuration rather than the steric hindrance of anion ligands. As shown in Table S2, the distortion of the two binding modes of ligands in complex **2** is slightly greater than that in complex **1**, so the horizontal Zn···Zn distances of complex **2** are a little longer than those of complex **1**. The horizontal Zn···Zn distances of complex **3** are inconsistent with those in complexes **1** and **2**, which the big anion ligands, I, cause the configurations of two binding modes of ligands in complex **3** to change more greatly. As mentioned above, the structure of complex **3** is slightly different to those of complexes **1** and **2**. In complex **4**, the bigger anion ligand has been introduced, and the larger space resistance makes the horizontal and longitudinal Zn···Zn distances longer than those of complexes **1**–**3**. Furthermore, the ligand only adopts one binding mode and has three types of configuration, with the bigger anion ligand induced. This makes the complex **4** to be 2D network polymer and is not consistent with complexes **1**–**3**, the 1D polymer chains.

According above analysis, the structure of complexes have changed from 1D polymer chain to 2D network polymer under the similar reaction condition with adjustment of the anion ligands. That is, the appropriate anion ligands can effectively modulate the structure of the complexes for this type of ligand due to the ligand flexibility in binding modes and configurations.

### 3.3. UV-Vis Absorption and Optical Band Gap

**Figure 8:**
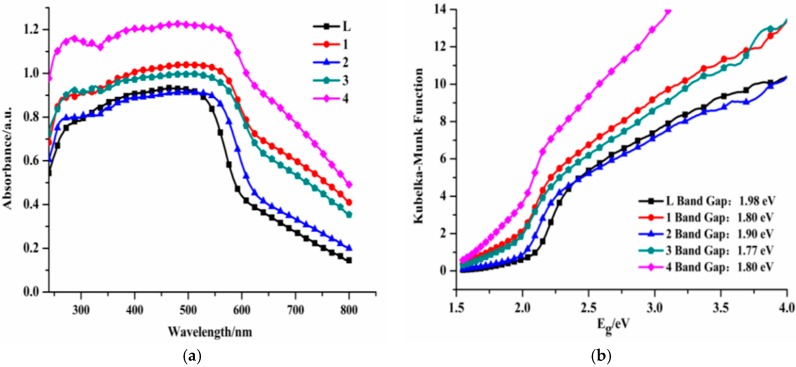
Solid state UV-vis absorption spectra (**a**) and Kubelka-Munk diffuse reflectance spectra (**b**) for **L** and complexes **1**–**4**.

The solid state UV-vis absorption spectra for **L** and complexes **1**–**4** have been carried out at room temperature. As shown in Figure 8a, the ligand exhibits a broad absorption in the 280–580 nm. Complexes **1**–**4** have similar absorption spectra compared with the free ligand. They are broader than those of the ligand and extend to near 650 nm, which is because a metal-ligand charge transition (**MLCT**) exists in the complexes [21]. The diffuse reflectivity for powder samples was measured to investigate the conductivity of the ligand and the four complexes and to obtain their band gaps (***E_g_***). The band gaps (***E_g_***) have been confirmed as the intersection point between the axis and the line extrapolated from the linear portion of the adsorption edge in a plot of the Kubella-Munk function ***F*** against ***E*** [21,24], which are shown in Figure 8b. The reflectance results show the presence of optical gaps, ***E_g_*** ≈ 1.98 (**L**), 1.80 (**1**), 1.90 (**2**), 1.77 (**3**), and 1.80 (**4**) eV, which suggest that the ligand and complexes **1**–**4** may have semiconductor properties, which have potential applications in the field of semiconductor materials.

## 4. Conclusions

In summary, the four novel Zn^II^ complexes, [(ZnCl_2_)_2_(L)_2_]_n_ (**1**), [(ZnBr_2_)_2_(L)_2_]_n_ (**2**) and [(ZnI_2_)_2_(L)_2_]_n_ (**3**) and {[Zn(SCN)_2_]_1.5_(L)_3_}_n_ (**4**), have been synthesized with the ligand, 4′-ferrocenyl-4,2′:6′,4′′-terpyridine. The X-ray structural analysis shows that complexes **1**–**3** are of 1D polymer chains and complex **4** is of 2D network polymer, which indicates that the selection of coordination anions can modulate the structure of complexes with this type of ligand under a similar reaction condition. Furthermore, the optical band gaps can be measured by the Kubelka-Munk-transformed diffuse reflectance spectra, and the related data show that the ligand and complexes **1**–**4** possess semiconductivity properties, which have potential applications in the field of semiconductor materials.

## Figures and Tables

**Table 1:** Crystallographic data and structural refinement parameters for **L** and complexes **1**–**4**.

Compounds	L	1	2	3	4
Empirical Formula	C_25_H_19_FeN_3_	C_50_H_38_Cl_4_Fe_2_ N_6_O_3_Zn_2_	C_51_H_46_Br_4_Fe_2_N_6_ O_3_Zn_2_	C_50_H_38_Fe_2_I_4_N_6_Zn_2_	C_162_H_120_Cl_18_Fe_6_ N_24_S_6_Zn_3_
Formula Weight	417.28	1155.10	1353.02	1472.90	3764.49
Crystal System	Orthorhombic	Triclinic	Triclinic	Triclinic	Monoclinic
Space Group	P2_1_2_1_2_1_	Pī	Pī	Pī	P2_1_/n
*a*(Å)	10.027(8)	8.700(5)	8.901(5)	9.1633(18)	15.271(5)
*b*(Å)	11.335(9)	16.897(5)	17.336(5)	16.802(3)	16.110(5)
*c*(Å)	16.980(14)	17.913(5)	18.223(5)	18.237(4)	32.652(5)
*a*[°]	90.00	76.423(5)	72.998(5)	71.159(2)	90.000(5)
*b*[°]	90.00	83.211(5)	80.299(5)	77.289(2)	100.019(5)
*γ*[°]	90.00	85.013(5)	82.664(5)	81.326(2)	90.000(5)
*V*(Å^3^)	1930(3)	2537.0(18)	2641.4(18)	2582.4(9)	7910(4)
*Z*	4	2	2	2	2
*R*_1_, *wR*_2_	0.0372	0.0477	0.0589	0.0369	0.0679
[*I ≥ 2σ* (*I*)]	0.0999	0.1481	0.1684	0.1127	0.1778
*R*_1_, *wR*_2_	0.0447	0.0657	0.0978	0.0512	0.0890
[*all data*]	0.1045	0.1640	0.1867	0.1225	0.1960
S on *F*^2^	1.068	1.071	1.068	1.057	1.049
CCDC	1,012,997	1,407,410	1,045,348	1,439,156	1,407,959

## Supplementary Materials

The following are available online at www.mdpi.com/1996-1944/10/12/1360/s1. Full crystallographic data in CIF format for ligand and complexes **1**–**4** have been deposited with the Cambridge crystallographic Data Centre, with CCDC No. 1012997 (**L**), 1407410 (**1**), 1045348 (**2**), 1439156 (**3**), and 1407959 (**4**). Copies of the data can be obtained free of charge, on application to CCDC, 12 Union Road, Cambridge CB2 1EZ, UK; Fax: +44(0)-1223-336033; or email: deposit@ccdc.cam.ac.uk. Table S1. Selected bond parameters of complexes **1**–**3**, Table S2. Collection distances of rings and dihedral angles for free ligand and complexes **1**–**4**, Table S3. Selected bond parameters of complex **4**.

## References

[1] Housecroft C.E. (2014). 4,2′:6′,4″-Terpyridines: Diverging and diverse building blocks in coordination polymers and metallomacrocycles. Dalton Trans..

[2] Housecroft C.E. (2015). Divergent 4,2′:6′,4′- and 3,2′:6′,3″-terpyridines as linkers in 2- and 3-dimensional architectures. CrystEngComm.

[3] Schubert U.S., Hofmeier H., Newkome G.R. (2006). Modern Terpyridine Chemistry.

[4] Klein Y.M., Prescimone A., Constable E.C., Housecroft C.E. (2017). 4,2′:6′,4″- and 3,2′:6′,3″-Terpyridines: The Conflict between Well-Defined Vectorial Properties and Serendipity in the Assembly of 1D-, 2D- and 3D-Architectures. Materials.

[5] Khatua S., Goswami S., Biswas S., Tomar K., Jena H.S., Konar S. (2015). Stable Multiresponsive Luminescent MOF for Colorimetric Detection of Small Molecules in Selective and Reversible Manner. Chem. Mater..

[6] Chen N., Li M.X., Yang P., He X., Shao M., Zhu S.R. (2013). Chiral Coordination Polymers with SHG-Active and Luminescence: An Unusual Homochiral 3D MOF Constructed from Achiral Components. Cryst. Growth Des..

[7] Schubert U.S., Winter A., Newkome G.R. (2011). Terpyridine-Based Materials.

[8] Constable E.C. (2007). 2,2′:6′,2″-terpyridines: From chemical obscurity to common supramolecular motifs. Chem. Soc. Rev..

[9] Butler I.R., McDonald S.J., Hursthouse M.B., Abdul Malik K.M. (1995). Ferrocenylpyridines: A new synthesis of 4′-ferrocenylterpyridine and the single crystal structure of a C_3_-ferrocenophane, [(*η*-C_5_H_4_CHCH_2_C(O)2-C_5_H_4_N)_2_CHC(O)2-C_5_H_4_N]Fe. Polyhedron.

[10] Barquín M., Cancela J., González Garmendia M.J., Quintanilla J., Amador U. (1998). Coordination compounds of 4,2′-6′,4″-terpyridine, [MCl_2_(4,2′-6′,4″-terpyridine)], M = Mn (II), Co (II), Ni (II), Cu (II) or Zn (II). Crystal structure of catena-poly [(dichlorozinc)-*μ*-(4,2′-6′,4″-terpyridine)]. Polyhedron.

[11] Constable E.C., Housecroft C.E., Neuburger M., Vujovic S., Zampese J.A., Zhang G. (2012). Cobalt (II) coordination polymers with 4′-substituted 4,2′:6′,4″- and 3,2′:6′,3″-terpyridines: Engineering a switch from planar to undulating chains and sheets. CrystEngComm.

[12] Nijs T., Malzner F.J., Fatayer S., Wackerlin A., Nowakowska S., Constable E.C., Housecroft C.E., Jung T.A. (2015). Programmed assembly of 4,2′:6′,4″-terpyridine derivatives into porous, on-surface networks. Chem. Commun..

[13] Maximilian Klein Y., Constable E.C., Housecroft C.E., Prescimone A. (2015). A 3-dimensional {4^2^·8^4^}lvt net built from a ditopic bis(3,2′:6′,3″-terpyridine) tecton bearing long alkyl tails. CrystEngComm.

[14] Granifo J., Vargas M., Garland M.T., Ibáñez A., Gaviño R., Baggio R. (2008). The novel ligand 4′-phenyl-3,2′:6′,3″-terpyridine (L) and the supramolecular structure of the dinuclear complex [Zn_2_(*μ*-L)(acac)_4_]·H_2_O (acac = acetylacetonato). Inorg. Chem. Commun..

[15] Wang B.C., Chen X.L., Hu H.M., Yao H.L., Xue G.L. (2009). Two novel Zn (II) coordination polymers based on trigonal ligand: 4′-(4-pyridyl)-3,2′:6′,3″-terpyridine. Inorg. Chem. Commun..

[16] Yang P., Wang M.S., Shen J.J., Li M.X., Wang Z.X., Shao M., He X. (2014). Seven novel coordination polymers constructed by rigid 4-(4-carboxyphenyl)-terpyridine ligands: Synthesis, structural diversity, luminescence and magnetic properties. Dalton Trans..

[17] Zhao M., Tan J., Su J., Zhang J., Zhang S., Wu J., Tian Y. (2016). Syntheses, crystal structures and third-order nonlinear optical properties of two series of Zn (II) complexes using the thiophene-based terpyridine ligands. Dyes Pigments.

[18] Klein Y.M., Prescimone A., Constable E.C., Housecroft C.E. (2016). Coordination Behaviour of 1-(4,2′:6′,4″-terpyridin-4′-yl) ferrocene and 1-(3,2′:6′,3″-terpyridin-4′-yl) ferrocene: Predictable and Unpredictable Assembly Algorithms. Aust. J. Chem..

[19] Zhang L., Li C.J., He J.E., Chen Y.Y., Zheng S.R., Fan J., Zhang W.G. (2016). Construction of New Coordination Polymers from 4′-(2,4-disulfophenyl)-3,2′:6′,3″-terpyridine: Polymorphism, pH-dependent syntheses, structures, and properties. J. Solid State Chem..

[20] Wang X.C., Tian Y.P., Kan Y.H., Zuo C.Y., Wu J.Y., Jin B.K., Zhou H.P., Yang J.X., Zhang S.Y., Tao X.T. (2009). Functionalized ferrocenes and ferroceniums: Synthesis, crystal structures and electrochemical properties based on carbazole/phenothiazine-ferrocene conjugated molecules. Dalton Trans..

[21] Xiao L.F., Zhu L.Y., Zeng Q.L., Liu Q.S., Zhang J., Li S.L., Zhou H.P., Zhang S.Y., Wu J.Y., Tian Y.P. (2015). Novel metal-organic hybrid materials constructed by ferrocenyl terpyridine derivatives and Zn^II^X_2_ (X = Cl^−^, Br^−^, I^−^, SCN^−^ and CH_3_COO^−^). J. Organomet. Chem..

[22] Farlow B., Nile T.A., Walsh J.L., McPhail A.T. (1993). Synthesis, X-ray structural determination and coordination chemistry of 4′-Ferrocenyl-2,2′:6′,2″-terpyridine. Polyhedron.

[23] Constable E.C., Edwards A.J., Martinez-Manez R., Raithby P.R., Thompson A.M.C. (1994). Complexes containing Ferrocenyl Groups as Redox Spectators; Synthesis, Molecular Structure and Coordination Behaviour of 4′-Ferrocenyl-2,2′:6′,2″-terpyridine. J. Chem. Soc. Dalton Trans..

[24] Liu B., Yang J., Yang G.C., Ma J.F. (2013). Four new three-dimensional polyoxometalate-based metal-organic frameworks constructed from[Mo_6_O_18_(O_3_AsPh)_2_^4−^ polyoxoanions and copper(I)-organic fragments: Syntheses, structures, electrochemistry, and photocatalysis properties. Inorg. Chem..

