# Supplementary material for: Four Novel Zn (II) Coordination Polymers Based on 4′-Ferrocenyl-3,2′:6′,3′′-Terpyridine: Engineering a Switch from 1D Helical Polymer Chain to 2D Network by Coordination Anion Modulation
Source: Materials (Basel). 2017 Nov 27;10(12):1360. doi: 10.3390/ma10121360 (PMC5744295; doi:10.3390/ma10121360)
Supplement: Supplementary file 1 [file materials-10-01360-s001.pdf]

Supplementary Materials: Four Novel Zn (II) Coordination Polymers Based on 4'-Ferrocenyl-3,2':6',3''-Terpyridine: Engineering a Switch from 1D Helical Polymer Chain to 2D Network by Coordination Anion Modulation

Lufei Xiao, Dajun Wu, Xuchun Wang, Wei Du, Jun Zhang, Shengli Li, Hongping Zhou, Jieying Wu and Yupeng Tian

Table S1. Selected bond parameters of complexes **1**–**3**.

Compounds	Bond Parameters			
	**Bond Distance/Å**			
1	Zn1-Cl1	2.2067(15)	Zn2-Cl3	2.1983(16)
	Zn1-Cl2	2.2057(17)	Zn2-Cl4	2.2178(14)
	Zn1-N1	2.063(4)	Zn2-N3	2.046(4)
	Zn1-N6	2.051(4)	Zn2-N4	2.059(3)
	**Bond Angle/°**			
	N1-Zn1-N6	97.08(16)	N3-Zn2-N4	105.13(15)
	N1-Zn1-Cl1	107.27(11)	N3-Zn2-Cl3	107.77(11)
	N1-Zn1-Cl2	110.65(11)	N3-Zn2-Cl4	106.39(11)
	N6-Zn1-Cl1	111.83(11)	N4-Zn2-Cl3	109.88(11)
	N6-Zn1-Cl2	107.37(11)	N4-Zn2-Cl4	107.86(10)
	Cl1-Zn1-Cl2	120.19(8)	Cl3-Zn2-Cl4	118.93(8)
	**Bond Distance/Å**			
2	Zn1-Br1	2.3591(12)	Zn2-Br3	2.3443(13)
	Zn1-Br2	2.3485(13)	Zn2-Br4	2.3481(13)
	Zn1-N1	2.055(6)	Zn2-N3	2.088(5)
	Zn1-N6	2.053(6)	Zn2-N4	2.079(6)
	**Bond Angle/°**			
	N1-Zn1-N6	106.2(2)	N3-Zn2-N4	96.9(2)
	N1-Zn1-Br1	110.03(16)	N3-Zn2-Br3	111.77(14)
	N1-Zn1-Br2	109.23(16)	N3-Zn2-Br4	107.67(14)
	N6-Zn1-Br1	105.27(16)	N4-Zn2-Br3	108.77(16)
	N6-Zn1-Br2	106.28(15)	N4-Zn2-Br4	110.01(17)
	Br1-Zn1-Br2	118.96(6)	Br3-Zn2-Br4	119.41(6)
	**Bond distance/Å**			
3	Zn1-I1	2.5322(8)	Zn2-I3	2.5465(8)
	Zn1-I2	2.5491(8)	Zn2-I4	2.5547(9)
	Zn1-N1	2.042(4)	Zn2-N3	2.070(4)
	Zn1-N6	2.050(4)	Zn2-N4	2.065(4)
	**Bond Angle/°**			
	N1-Zn1-N6	106.72(17)	N3-Zn2-N4	100.46(17)
	N1-Zn1-I1	109.92(12)	N3-Zn2-I3	108.84(12)
	N1-Zn1-I2	109.77(12)	N3-Zn2-I4	109.21(12)
	N6-Zn1-I1	106.62(11)	N4-Zn2-I3	110.21(11)
	N6-Zn1-I2	103.42(11)	N4-Zn2-I4	106.57(13)
	I1-Zn1-I2	119.46(3)	I3-Zn2-I4	119.81(3)

Table S2. Collection distances of rings and dihedral angles for free ligand and complexes **1**–**4**.

Compounds	Binding Mode	Planes	Collection Distance/ Å	Dihedral Angles/°
L		P1/P2	-	1.532
		P2/P4	1.464 (C8-C20)	28.650
		P4/P5	1.474 (C10-C11)	35.331
		P3/P4	1.486 (C4-C6)	1.908
1	Binding Mode II	P1/P2		4.137
		P2/P4	1.484 (C8-C20)	40.116
		P4/P5	1.490 (C10-C11)	23.814
		P3/P4	1.493 (C4-C6)	28.990
	Binding Mode III	P6/P7	-	1.984
		P7/P9	1.464 (C33-C45)	5.275
		P9/P10	1.487 (C35-C36)	33.248
		P8/P9	1.485 (C29-C31)	8.719
2	Binding Mode II	P1/P2		3.221
		P2/P4	1.496 (C35-C36)	40.984
		P4/P5	1.486 (C38-C39)	26.208
		P3/P4	1.472 (C45-C46)	26.576
	Binding Mode III	P6/P7	-	1.638
		P7/P9	1.497 (C10-C11)	7.403
		P9/P10	1.510 (C20-C21)	35.260
		P8/P9	1.499 (C13-C14)	7.056
3	Binding Mode II	P1/P2	-	1.974
		P2/P4	1.463(C33-C45)	30.372
		P4/P5	1.508(C35-C36)	30.853
		P3/P4	1.496(C29-C31)	16.891
	Binding Mode III	P6/P7	-	1.329
		P7/P9	1.471 (C8-C20)	17.979
		P9/P10	1.493 (C10-C11)	25.945
		P8/P9	1.496 (C2-C6)	4.523
4	Binding mode II-1	P1/P2	-	1.767
		P2/P4	1.482 (C7-C11)	17.386
		P4/P5	1.494 (C13-C49)	45.571
		P3/P4	1.484 (C15-C16)	50.153
	Binding mode II-2	P6/P7	-	1.167
		P7/P9	1.477 (C28-C31)	2.602
		P9/P10	1.487 (C29-C41)	43.196
		P8/P9	1.496 (C25-C26)	33.348
	Binding mode II-3	P11/P12	-	4.401
		P12/P14	1.472 (C64-C66)	6.291
		P14/P15	1.487 (C57-C58)	31.672
		P13/P14	1.487 (C54-C56)	35.908

Table S3. Selected bond parameters of complex **4**.

**Bond distance/Å**			
Zn1- N1	2.290(4)	Zn1-N10	2.058(4)
Zn1-N3	2.309(4)	Zn2-N7	2.279(4)
Zn1-N5	2.249(4)	Zn2-N18	2.272(4)
Zn1-N8	2.288(4)	Zn2-N11	2.079(4)
Zn1-N9	2.062(4)	-	-
**Bond angle/°**			
N1-Zn1-N3	179.35(15)	N5-Zn1-N10	91.12(16)
N1-Zn1-N5	93.78(15)	N8-Zn1-N9	89.68(16)
N1-Zn1-N8	86.13(15)	N8-Zn1-N10	89.07(17)
N1-Zn1-N9	89.72(16)	N9-Zn1-N10	178.47(18)
N1-Zn1-N10	91.08(16)	N7-Zn2-N18	87.82(15)
N3-Zn1-N5	86.72(16)	N7-Zn2-N18 [a]	92.18(15)
N3-Zn1-N8	93.36(15)	N7-Zn2-N11	90.24(16)
N3-Zn1-N9	89.86(16)	N7-Zn2-N11 [a]	89.76(16)
N3-Zn1-N10	89.32(16)	N11-Zn2-N18	91.02(16)
N5-Zn1-N8	179.79(16)	N11-Zn2-N18 [a]	88.98(16)
N5-Zn1-N9	90.13(16)	-	-

[a] Symmetry transformations used to generate the equivalent atoms: -x, -y, 1-z.

© 2017 by the authors. Submitted for possible open access publication under the terms and conditions of the Creative Commons Attribution (CC BY) license (http://creativecommons.org/licenses/by/4.0/).
